# Association Between Active Commuting and Incident Cardiovascular Diseases in Chinese: A Prospective Cohort Study

**DOI:** 10.1161/JAHA.119.012556

**Published:** 2019-10-02

**Authors:** Mengyu Fan, Jun Lv, Canqing Yu, Yu Guo, Zheng Bian, Songchun Yang, Ling Yang, Yiping Chen, Yuelong Huang, Biyun Chen, Lei Fan, Junshi Chen, Zhengming Chen, Lu Qi, Liming Li, the China Kadoorie Biobank Collaborative Group

**Affiliations:** ^1^ Department of Epidemiology and Biostatistics School of Public Health Peking University Health Science Center Beijing China; ^2^ Department of Epidemiology School of Public Health and Tropical Medicine Tulane University New Orleans LA; ^3^ Key Laboratory of Molecular Cardiovascular Sciences (Peking University) Ministry of Education Beijing China; ^4^ Peking University Institute of Environmental Medicine Beijing China; ^5^ Chinese Academy of Medical Sciences Beijing China; ^6^ Clinical Trial Service Unit & Epidemiological Studies Unit (CTSU) Nuffield Department of Population Health University of Oxford United Kingdom; ^7^ Hunan Center for Disease Control and Prevention Hunan China; ^8^ Henan Center for Disease Control and Prevention Henan China; ^9^ China National Center for Food Safety Risk Assessment Beijing China; ^10^ Department of Nutrition Harvard School of Public Health Boston MA

**Keywords:** physical activity, commuting, cycling, walking, cardiovascular disease, Cardiovascular Disease, Epidemiology, Primary Prevention, Lifestyle

## Abstract

**Background:**

Active commuting is related to a higher level of physical activity but more exposure to ambient air pollutants. With the rather serious air pollution in urban China, we aimed to examine the association between active commuting and risk of incident cardiovascular disease in the Chinese population.

**Methods and Results:**

A total of 104 170 urban commuters without major chronic diseases at baseline were included from China Kadoorie Biobank. Self‐reported commuting mode was defined as nonactive commuting, work at home or near home, walking, and cycling. Multivariable Cox regression was used to examine associations between commuting mode and cardiovascular disease. Overall, 47.2% of the participants reported nonactive commuting, 13.4% reported work at home or work near home, 20.1% reported walking, and 19.4% reported cycling. During a median follow‐up of 10 years, we identified 5374 incidents of ischemic heart disease, 664 events of hemorrhagic stroke, and 4834 events of ischemic stroke. After adjusting for sex, socioeconomic status, lifestyle factors, sedentary time, body mass index, comorbidities, household air pollution, passive smoking, and other domain physical activity, walking (hazard ratio, 0.90; 95% CI, 0.84–0.96) and cycling (hazard ratio, 0.81; 95% CI, 0.74–0.88) were associated with a lower risk of ischemic heart disease than nonactive commuting. Cycling was associated with a lower risk of ischemic stroke (hazard ratio, 0.92; 95% CI, 0.84–1.00). No significant association was found of walking or cycling with hemorrhagic stroke. The associations of commuting mode with major cardiovascular disease were consistent among men and women and across different levels of other domain physical activity.

**Conclusions:**

In urban China, cycling was associated with a lower risk of ischemic heart disease and ischemic stroke. Walking was associated with a lower risk of ischemic heart disease.

**Clinical Perspective**

**What Is New?**

- Little is known whether the long‐term benefits of active commuting on cardiovascular health still hold in China, where air pollution is severer than the developed countries.
- In this prospective cohort study of Chinese urban adults, commuting by cycling was associated with a reduced risk of ischemic heart disease and ischemic stroke; and walking was associated with a reduced risk of ischemic heart disease.
- The protective effects of cycling and walking commuting were persistent in populations of different characteristics.

**What Are the Clinical Implications?**

- Our findings support public health efforts to encourage adults to adopt a more active mode of commuting, particularly by cycling, to deliver cardiovascular benefits at the population level.

## Introduction

Insufficient physical activity is one of the leading risk factors for cardiovascular diseases (CVDs). Active commuting such as walking and cycling to and from work has been advocated to improve physical activity. Active commuters, however, had the highest inhalation and uptake dose of air pollutants because of increased inhalation rates,^1^ which may counteract its beneficial effects on cardiovascular health.

Evidence for the long‐term effects of active commuting on CVDs has not been consistent.^2^, ^3^, ^4^, ^5^, ^6^, ^7^ A recent study of 263 540 participants from UK Biobank reported that commuting by cycling and walking were associated with a lower risk of CVD incidence.^8^ However, the previous work was limited by several potential biases, for example, no adjustment for overall noncommuting activity level and no differentiation between different cardiovascular end points (ischemic heart disease [IHD] and stroke). More importantly, little is known regarding whether such long‐term protective effects persist in other nonwhite populations from developing countries, like the Asian population, where air pollution is severer than in the developed countries. Therefore, in the present study of China Kadoorie Biobank (CKB), we aimed to examine the association between active commuting and risk of incident CVDs, which combines counteracting effects of physical activity and air pollution, in the Chinese population.

## Methods

### Study Design

Details of how to access CKB data and details of the data release schedule are available online.^9^ The CKB is a large, population‐based prospective cohort of >0.5 million Chinese adults. Details of the study design and sample characteristics have been described previously.^10^, ^11^ Briefly, the CKB study took place in 10 geographically defined regions (5 urban and 5 rural) of China between 2004 and 2008. These regions were chosen according to local disease patterns, exposure to certain risk factors, population stability, quality of death and disease registries, local commitment, and capacity. All registered residents aged 35 to 74 years were identified through official residential records, then invited to participate in the baseline survey; ≈30% of those invited responded (33% [26%–38%] in rural areas and 27% [16%–50%] in urban areas). Overall, a total of 512 715 men and women (including a few slightly outside the target age range) were recruited. At the baseline survey, trained health workers collected detailed information using an interview‐administered laptop‐based questionnaire; took physical measurements; and collected blood samples for long‐term storage. All participants provided written informed consent. The Ethical Review Committee of the Chinese Center for Disease Control and Prevention (Beijing, China) and the Oxford Tropical Research Ethics Committee, University of Oxford (UK) approved the study.

### Assessment of Physical Activity

The baseline questions on physical activity were adapted from validated questionnaires as previously described.^12^, ^13^ We asked participants about their usual type and duration for each of the 4 domains of physical activity (occupational, commuting, household, and leisure time) during the past year. The updated 2011 Compendium of Physical Activities was used to assign an intensity level of each activity.^14^ The physical activity level of each activity was calculated by multiplying its assigned metabolic equivalent value by hours spent on that activity per day.

For commuting behavior, there were 2 sets of commuting‐related questions: nonfarmers employed at baseline were asked, “In the past 12 months, how did you usually get to and from work?” with the responses being: (1) mainly walked; (2) by bicycle; (3) motorcycle or moped; (4) by car or by bus/ferry/train; (5) mainly work at home or work near home. Daily commuting time (in minutes) was also recorded, except participants who responded “mainly work at home or work near home.” For “farmers,” however, only 1 related question was asked: “How many minutes do you usually spent walking or cycling to and from work on a typical day?.” Thus, we restricted our analyses to nonfarmers from 5 urban areas, among whom complete commuting information was collected.

We derived 4 commuting categories: nonactive (motorcycle or moped, by car, or by bus/ferry/train); mainly work at home or work near home; walking; and cycling. For walking and cycling, we further categorized participants into 4 groups according to the daily commuting time reported by the participant: <15, 15 to 29, 30 to 59, and ≥60 minutes/day.

After completion of the baseline survey, we randomly selected about 5% of the surviving participants in 10 areas for the first resurvey during 2008. To test the reproducibility of the reported commuting time, total physical activity level, and transport mode, we analyzed 1300 participants who completed the same questionnaire twice at a median interval of 1.4 years. The respective intraclass correlation coefficient between the 2 questionnaires was 0.42 for commuting time and 0.59 for total physical activity level, and the classification agreement was 84.3% (κ=0.49; *P*<0.001) for commuting mode.

### Assessment of Covariates

Covariate information collected in the baseline questionnaire included sociodemographic characteristics (age, sex, education, marital status, household income, and occupation), lifestyle behaviors (alcohol consumption, smoking status, and intakes of red meat, fresh fruits, and vegetables, leisure sedentary time), passive smoking, household air pollution (cooking pollution and heating pollution), personal health and medical history (hypertension and diabetes mellitus), and family histories of heart attack or stroke.

Trained staff undertook baseline measurements of body weight, height, and blood pressure by using calibrated instruments. Body mass index (BMI) was calculated as measured weight in kilograms divided by height in meters squared. Prevalent hypertension was defined as a measured systolic blood pressure of 140 mm Hg or more, a measured diastolic blood pressure of 90 mm Hg or more, self‐reported diagnosis of hypertension, or self‐reported use of antihypertensive drugs at baseline. Prevalent diabetes mellitus was defined as measured fasting blood glucose ≥7.0 mmol/L, measured random blood glucose ≥11.1 mmol/L, or self‐reported diagnosis of diabetes mellitus.

### Ascertainment of Study Outcomes

We collected participants’ status periodically through local disease and death registries, as well as the national health insurance system.^15^ Electronic linkage with the health insurance system is carried out every 6 months in each region. Almost all of the study participants (≈98%) had been successfully linked to the health insurance databases. If participants failed to be linked, active confirmation of status was conducted annually by local street committees or village administrators. Fatal and nonfatal events were coded using the *International Classification of Diseases, Tenth Revision* (*ICD‐10*) by trained staff “blinded” to baseline exposures. The primary study outcomes were the incidence of the following diseases: IHD (I20‐I25), hemorrhagic stroke (I61), and ischemic stroke (IS, I63).

### Statistical Analysis

For the present analysis, we included nonfarmers from 5 urban areas (n=217 076), among whom complete commuting information was collected. We further excluded participants who reported no work (n=110 670); those who reported a history of heart disease (n=10 453), stroke (n=5241), or cancer (n=1390) at baseline; and those who were recorded with an implausible censoring date for loss to follow‐up (n=1), leaving 104 170 participants for the final analyses. Analyses were conducted with Stata (version 13.0, StataCorp, College Station, TX). Statistical tests were 2‐sided, and *P*<0.05 was considered to indicate statistical significance.

Person‐years at risk were calculated from the recruitment date at baseline to the date of study outcome diagnosis, death, loss to follow‐up, or December 31, 2016, whichever occurred first. By December 31, 2016, 4781 (<1%) participants were lost to follow‐up. Stratified Cox regression was used to estimate hazard ratios (HRs) and 95% CIs for incident risks of major CVDs associated with baseline commuting mode, with stratification on age at risk (5‐year intervals) and study area. The use of age at risk and study area–stratified Cox models is a standard method used in the CKB. Stratification allows for different baseline hazard function for each stratum.^16^ The proportional hazards assumption for the stratified Cox model was checked by a test and graph based on Schoenfeld residuals and the proportional hazards assumption was satisfied. The reference category for all analyses was nonactive commuting, in line with previous analyses of the United Kingdom. Models were adjusted for sex, education (no formal school, primary school, middle school, high school, or college or university or higher); marital status (married, widowed, divorced/separated, or never married); household income (<10 000, 10 000–19 999, ≥20 000 Chinese renminbi/year); occupation (factory worker, administrator/manager/professional/technical, sales/service workers/self‐employed, or others); alcohol consumption (less than weekly; former regular drinkers; weekly; or <15, 15–29, 30–59, or ≥60 g/d of pure alcohol); smoking status (never/occasional smokers, former smokers who had stopped smoking for reasons other than illness for ≥6 months, current smokers and former smokers who stopped smoking because of illness: 1–14, 15–24, or ≥25 cigarettes or equivalents/day); intake frequencies of red meat, fresh fruits, and vegetables (daily, 4–6 d/wk, 1–3 d/wk, monthly, or rarely or never); leisure sedentary time (in hours per day); family histories of heart attack or stroke (yes or no, only in the corresponding analysis); BMI (in kilograms per meter squared); prevalent hypertension (yes or no); prevalent diabetes mellitus (yes or no); cooking pollution (never or rarely cooking; cooking with clean fuels, solid fuels, or other fuels); heating pollution (no winter heating; winter heating with clean fuels, solid fuels, or other fuels); passive smoking (no exposure, lived with smoker <20 years, lived with smoker ≥20 years and current exposure <20 h/wk, lived with smoker ≥20 years and current exposure ≥20 h/wk); and work, housework, and leisure time–specific physical activity level (in metabolic equivalents, hours per day). We further examined the associations of daily walking and cycling time with incident risks of major CVDs on the basis of the multivariable adjusted Cox models (<15, 15–29, 30–59, and ≥60 min/d compared with nonactive commuting, respectively). To test the linear trend across categories, we assigned the median value to each commuting time category and then treated the variable as continuous in a separate Cox model.

To examine whether the association of commuting mode with major CVDs differed by baseline characteristics, subgroup analyses were conducted to test for interaction of commuting mode with 13 baseline factors: sex (men or women), age (<50 or ≥50 years), education (illiterate/primary school or middle school and above), marital status (unmarried or married), household income (<20 000 or ≥20 000 Chinese renminbi/y), occupation (factory worker or not), smoking status (current daily smoker or not), alcohol consumption (current weekly drinker or not), other domain physical activity level (categorized using tertile cutoffs), leisure sedentary time (<3 or ≥3 h/d), BMI (<24.0, 24.0–27.9, or ≥28.0), hypertension (yes or no), and diabetes mellitus (yes or no). In the subgroup and interaction analyses, stratified Cox models were used and analyses were done separately for each baseline characteristic. The tests for interaction were performed by means of likelihood ratio tests, which involved comparing models with and without cross product terms between the baseline characteristic and categories of commuting mode. We further conducted sensitivity analyses to reduce the potential impact of reverse causation by excluding events occurring during the first 2 years of follow‐up.

## Results

### Characteristics of Study Participants

Among the 104 170 commuters analyzed, the mean age was 45.9 years and 48.6% were women. Overall, 47.2% of the participants reported nonactive commuting, 13.4% reported work at home or work near home, 20.1% reported walking, and 19.4% reported cycling, respectively (Table 1). In general, active commuters were, on average, older, more likely to be women, had lower education level and household income, had a higher level of other domain physical activity, and tended to use solid fuels for cooking and heating, compared with nonactive commuters.

**Table 1:** Baseline Characteristics of 104 170 Study Participants According to Baseline Commuting Mode

	Nonactive Commuting	Work at Home or Near Home	Walking	Cycling
Participants, n (%)	49 145 (47.2)	13 936 (13.4)	20 912 (20.1)	20 177 (19.4)
Age, y	44.3 (6.7)	49.1 (9.1)	45.9 (7.2)	47.7 (7.6)
Men, %	58.0	43.6	42.9	49.2
Middle school and above, %	83.1	77.4	80.8	76.6
Married, %	94.0	92.0	93.1	93.5
Household income ≥20 000 Chinese renminbi/y, %	77.1	62.4	66.2	59.7
Factory worker, %	49.8	25.8	47.5	61.0
Current weekly alcohol drinker, %	25.0	23.2	23.8	22.8
Current daily smoker, %	35.0	34.6	33.6	32.7
Average weekly consumption^a^				
Red meat, d/wk	5.3 (2.2)	5.0 (2.2)	5.1 (2.2)	5.0 (2.1)
Fresh vegetable, d/wk	6.9 (0.4)	6.9 (0.5)	6.9 (0.6)	6.9 (0.6)
Fresh fruit, d/wk	4.0 (2.4)	3.5 (2.6)	3.9 (2.6)	3.5 (2.6)
Other domain PA level, MET, h/d^b^	24.5 (10.9)	24.5 (10.9)	25.6 (10.8)	28.2 (10.8)
Leisure sedentary time, h/d	2.8 (1.3)	2.9 (1.4)	2.8 (1.3)	2.6 (1.4)
Family history of heart attack, %	4.8	4.2	4.8	4.3
Family history of stroke, %	20.0	18.8	19.9	20.0
Body mass index, kg/m^2^	24.2 (3.3)	24.2 (3.2)	24.0 (3.2)	23.8 (3.1)
Diabetes mellitus, %	4.4	4.8	4.3	3.6
Hypertension, %	24.6	26.0	25.5	24.0
Solid fuel use for cooking, %	3.1	4.9	3.8	5.5
Solid fuel use for heating, %	9.7	16.2	11.3	14.9
Secondhand smoking, %	88.2	88.0	86.8	87.8

Values are mean (standard deviation) or percentage. Values for age and sex were unadjusted, and those for other baseline characteristics were adjusted for age, sex, and study areas, using logistic regression (for categorical variables) or multiple linear regression (for continuous variables). Linear trend was assessed by assigning consecutive integers to 4 commuting mode categories in a separate model. All *P* values for trend were <0.003, except for family history of heart attack (*P*=0.035), family history of stroke (*P*=0.845), and hypertension (*P*=0.443). MET indicates metabolic equivalent; PA, physical activity.

a A short qualitative food frequency questionnaire was used to assess the habitual intakes of red meat, fresh vegetables, and fruits. Average weekly consumptions of red meat, fresh vegetables, and fruits were calculated by assigning participants to the midpoint of their consumption category.

b Other domain physical activity including occupational, housework, and leisure‐time physical activity.

### Association Between Commuting Mode and CVDs

The median follow‐up period was 9.9 years (total person‐years, 985 716) for incident CVD. Over the follow‐up period, there were 5374 events of IHD, 664 events of hemorrhagic stroke, and 4834 events of IS documented. Table 2 shows the associations between baseline commuting mode and prospective cardiovascular outcomes. After adjusting for sex, socioeconomic status, lifestyle factors, sedentary time, BMI, comorbidities, household air pollution, passive smoking, and other domain physical activity, work at home or near home (HR, 0.90; 95% CI, 0.82–0.99), walking (HR, 0.90; 95% CI, 0.84–0.96), and cycling (HR, 0.81; 95% CI, 0.74–0.88) were associated with a lower risk of IHD than nonactive commuting. For IS, an inverse association was observed for cycling (HR, 0.92; 95% CI, 0.84–1.00). Moreover, there was no significant association for work at/near home, walking, or cycling for hemorrhagic stroke. These associations were consistent across sex (all *P*>0.05 for heterogeneity; Figure, Tables S1 through Table S3).

**Table 2:** Adjusted Hazard Ratios for Cardiovascular Diseases by Baseline Commuting Mode

	Nonactive Commuting	Work at Home or Near Home	Walking	Cycling
Participants, n	49 145	13 936	20 912	20 177
Ischemic heart disease				
Cases	2524	717	1358	775
Incidence rate per 1000 PYs^a^	6.00	5.42	5.17	4.34
Sex‐adjusted	[Reference]	0.88 (0.81–0.97)	0.86 (0.81–0.92)	0.71 (0.66–0.77)
Multivariable‐adjusted^b^	[Reference]	0.90 (0.82–0.99)	0.90 (0.84–0.96)	0.80 (0.74–0.88)
Further adjusted for other domain PA^c^	[Reference]	0.90 (0.82–0.99)	0.90 (0.84–0.96)	0.81 (0.74–0.88)
Ischemic stroke				
Cases	1864	868	1243	859
Incidence rate per 1000 PYs^a^	4.82	5.48	5.02	4.43
Sex‐adjusted	[Reference]	1.11 (1.02–1.20)	1.05 (0.97–1.12)	0.90 (0.83–0.97)
Multivariable‐adjusted^b^	[Reference]	1.04 (0.95–1.13)	1.05 (0.97–1.13)	0.92 (0.84–1.00)
Further adjusted for other domain PA^c^	[Reference]	1.04 (0.95–1.13)	1.05 (0.97–1.13)	0.92 (0.84–1.00)
Hemorrhagic stroke				
Cases	266	126	122	150
Incidence rate per 1000 PYs^a^	0.60	0.82	0.57	0.68
Sex‐adjusted	[Reference]	1.30 (1.04–1.64)	0.94 (0.76–1.18)	1.11 (0.90–1.36)
Multivariable‐adjusted^b^	[Reference]	1.17 (0.92, 1.48)	0.90 (0.72–1.12)	1.03 (0.83–1.28)
Further adjusted for other domain PA^c^	[Reference]	1.17 (0.92–1.49)	0.89 (0.71–1.11)	1.01 (0.82–1.26)

Stratified Cox proportional models were used with stratification on age and study area. PA indicates physical activity; PY, person‐year.

a The incidence rate per 1000 person‐years was adjusted for age, sex, and study areas.

b Multivariable model was adjusted for sex, education; marital status; household income; occupation; alcohol consumption; smoking status; intake frequencies of red meat, fresh fruits, and vegetables; leisure sedentary time; family histories of heart attack or stroke (only in the corresponding analysis); body mass index; prevalent hypertension; prevalent diabetes mellitus; cooking pollution; heating pollution; and passive smoking.

c Other domain physical activity including occupational, housework, and leisure‐time physical activity (in metabolic equivalents, h/d).

**Figure 1:**
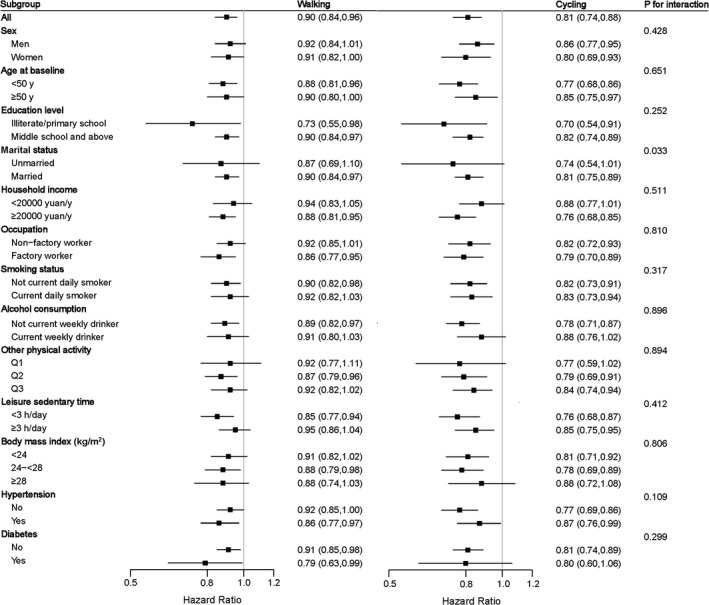
Subgroup analysis of associations between active commuting and ischemic heart disease according to potential baseline risk factors. The reference category for all analyses was nonactive commuting. Risk estimates for work at home or work near home are shown in Table S2. The black boxes represent hazard ratios, and the horizontal lines represent 95% CIs. Stratified Cox models were used and analyses were done separately for each baseline characteristic. Models were adjusted for sex; education; marital status; household income; occupation; alcohol consumption; smoking status; intake frequencies of red meat, fresh fruits, and vegetables; leisure sedentary time; family history of heart attack; body mass index; prevalent hypertension; prevalent diabetes mellitus; cooking pollution; heating pollution; passive smoking; and occupational, housework, and leisure‐time physical activity level, except for the stratified variable in the corresponding subgroup analysis.

Table 3 shows that among active commuters there were distinct dose‐response trends for IHD incidence by daily commuting time (*P*<0.001 for trend). For walking, the adjusted HRs for IHD were 1.00 (95% CI, 0.87–1.15), 0.95 (95% CI, 0.85–1.06), 0.87 (95% CI, 0.79–0.95), and 0.82 (95% CI, 0.71–0.95) among those who reported <15, 15 to 29, 30 to 59, and ≥60 minutes of walking compared with those who reported nonactive commuting. For cycling, the respective HRs were 0.85 (95% CI, 0.68–1.07), 0.73 (95% CI, 0.63–0.86), 0.82 (95% CI, 0.73–0.92), and 0.79 (95% CI, 0.67–0.92). There were no dose‐response trends for IS and hemorrhagic stroke incidence by daily active commuting time (Table 3).

**Table 3:** Adjusted Hazard Ratios for Incident Cardiovascular Diseases by Daily Walking and Cycling Time

	N	Cases	Incidence Rate^a^	HR (95% CI)	*P* for Trend
Ischemic heart disease					
Walking					
<15 min/d	3727	217	6.03	1.00 (0.87–1.15)	<0.001
15 to 29 min/d	6342	403	5.42	0.95 (0.85–1.06)	
30 to 59 min/d	8037	545	5.00	0.87 (0.79–0.95)	
≥60 min/d	2806	193	4.83	0.82 (0.71–0.95)	
Cycling					
<15 min/d	2364	76	4.20	0.85 (0.68–1.07)	<0.001
15 to 29 min/d	4999	178	3.63	0.73 (0.63–0.86)	
30 to 59 min/d	8103	339	4.01	0.82 (0.73–0.92)	
≥60 min/d	4711	182	3.73	0.79 (0.67–0.92)	
Ischemic stroke					
Walking					
<15 min/d	3727	194	4.98	1.04 (0.89–1.21)	0.520
15 to 29 min/d	6342	362	4.77	1.06 (0.95–1.19)	
30 to 59 min/d	8037	507	4.83	1.06 (0.96–1.17)	
≥60 min/d	2806	180	4.48	0.99 (0.85–1.16)	
Cycling					
<15 min/d	2364	83	3.79	0.91 (0.73–1.14)	0.059
15 to 29 min/d	4999	191	3.55	0.85 (0.73–0.99)	
30 to 59 min/d	8103	380	4.17	0.98 (0.87–1.10)	
≥60 min/d	4711	205	3.67	0.87 (0.74–1.01)	
Hemorrhagic stroke					
Walking					
<15 min/d	3727	19	0.49	0.78 (0.49–1.25)	0.233
15 to 29 min/d	6342	36	0.52	0.84 (0.59–1.20)	
30 to 59 min/d	8037	52	0.59	0.95 (0.70–1.29)	
≥60 min/d	2806	15	0.46	0.74 (0.43–1.25)	
Cycling					
<15 min/d	2364	18	0.72	1.02 (0.62–1.67)	0.303
15 to 29 min/d	4999	30	0.54	0.81 (0.55–1.19)	
30 to 59 min/d	8103	72	0.80	1.14 (0.87–1.51)	
≥60 min/d	4711	30	0.50	0.73 (0.49–1.09)	

The reference category for all analyses was nonactive commuting. Stratified Cox proportional models were used with stratification on age and study area. Models were adjusted for sex; education; marital status; household income; occupation; alcohol consumption; smoking status; intake frequencies of red meat, fresh fruits, and vegetables; leisure sedentary time; family history of heart attack or stroke (only in the corresponding analysis); body mass index; prevalent hypertension; prevalent diabetes mellitus; cooking pollution; heating pollution; passive smoking; occupational, housework, and leisure‐time physical activity level. HR indicates hazard ratio.

a The incidence rate per 1000 person‐years was adjusted for age, sex, and study areas.

In the sensitivity analyses of excluding events occurring during the first 2 years of follow‐up, similar associations were also observed.

### Subgroup Analyses

We further analyzed the associations between commuting mode and cardiovascular outcomes according to other potential baseline risk factors; associations were generally similar across subgroups stratified according to sex, age, education level, marital status, household income, occupation, smoking status, alcohol consumption, level of other domain physical activity, leisure sedentary time, BMI, diabetes mellitus, and hypertension (all *P*>0.05 for interaction, except for marital status: *P*=0.033 for interaction) (Figure, Tables S2 through S4).

## Discussion

In this large population‐based prospective study of Chinese, we found that daily commuting by walking and cycling, particularly those who commuted for a longer duration, were associated with lower risks of IHD. The protective effect on IHD was more robust from cycling than walking. In addition, cycling but not walking was associated with lower risk of IS. For hemorrhagic stroke, no significant association was found for walking or cycling. The associations of active commuting with major CVDs were consistent among men and women and across different levels of other domain physical activity.

Existing literature on active commuting mainly focused on CVD,^8^ coronary heart disease,^5^, ^6^ and cardiovascular risk factors.^17^, ^18^, ^19^ Our finding that active commuting was associated with a reduced risk of IHD was in agreement with these studies. The risk reduction was larger in the cycling group than in the walking group, reflecting the greater physiological intensity of cycling compared with walking.^8^ The associations between active commuting and IHD were independent of potential confounding factors such as sex, age, socioeconomic status, other lifestyle factors, sedentary time, BMI, and comorbidities, although inclusion of these variables reduced the strength of the association. Our findings were strengthened by the fact that there was a dose‐response relationship in reductions of IHD risk related to walking or cycling time spent commuting (minutes per day). The risk for developing IHD decreases as walking or cycling time increases. Taken together, active commuting, particularly cycling, is important to deliver IHD benefits among the Chinese population. To produce greater benefits, longer duration may be needed.

Intriguingly, we noted that the benefits of walking or cycling on IHD estimated in the present analysis were weaker than that previously reported in UK Biobank on CVD events (walking: HR, 0.73, 95% CI, 0.54–0.99; cycling: 0.54; 95% CI, 0.33–0.88). Several factors may account for this difference. First, it is more likely that cyclists are highly selected in the United Kingdom. Only 2.7% cycled in the United Kingdom, and they may be substantially different than the rest of the population in other aspects. Second, mixed‐mode commuting, such as walking to the bus, was included in the reference group, which may have led to an underestimation of the health effect of active commuting. Third, a more comprehensive adjustment was performed in this study. Moreover, the average level of active commuting as well as other domain physical activity, especially occupational physical activity, in the Chinese population is much higher, and there is evidence that the association of physical activity with CVD subtypes attenuated at the high level (ie, ≈20 metabolic equivalents, hours per day).^20^ In addition, such discrepancy might be partly attributable to the differed levels of air pollution between the United Kingdom and China.^21^ Few areas in China meet the air quality standards recommended by the World Health Organization.^22^ Previous studies found that exposures to air pollutants during active commuting were associated with acute adverse cardiovascular effects, including vascular impairment, arterial stiffness, and vascular reactivity.^23^, ^24^, ^25^ It is plausible that high exposure to air pollution during active commuting in high‐polluted areas may counteract the benefits of active commuting on cardiovascular health.

In addition, we found that work at home or work near home was associated with a lower risk of IHD compared with nonactive commuting, and the HR estimate of IHD associated with work at home or work near home was similar to that observed with walking. One possible explanation may be that those who work near home tend to walk to and from work and are also considered commute actively. As for those who work at home, they are considered nonactive, but they are less likely to be exposed to ambient air pollutants compared with nonactive commuters.

To our knowledge, only 1 study has assessed the association between active commuting and the risk of stroke. The study, conducted in Finland, which included 47 721 adults and documented 2863 incident stroke events, showed that walking or cycling to and from work was associated with a reduced risk of IS but not with hemorrhagic stroke.^26^ Consistent with previous results, we also found that cycling was associated with IS and not associated with hemorrhagic stroke. The reasons for the positive findings for IS versus the null finding for hemorrhagic stroke are unclear. It may be partly attributable to a relatively small number of events in the hemorrhagic stroke analyses, which may lead to limited power to detect an association. Future studies should further evaluate these associations.

The strengths of the present study included large sample size, a prospective cohort design, comprehensive information on both commuting and the other 3 domain‐specific physical activities, careful control for a wide range of established and potential confounders, and analyses of subtypes of CVDs. Because of the large sample size, we were able to examine the associations across population subgroups, which was not available in the previous studies. However, the present study had several limitations that need to be considered. Physical activity, including the mode and duration of commuting activity, was self‐reported. Although our questionnaire was shown to be reproducible, recall bias might have occurred. However, any bias introduced by misclassification of commuting behavior would probably lead to an underestimation of the association between active commuting and CVD outcomes^27^ We could not differentiate between car commuters and public transport commuters, and we combined public transport commuters into the reference category, which may have led to further underestimations of the health effects of active commuting. Moreover, we are unable to account for speed and distance of commuting, and commuting information was recorded at baseline and might not reflect the changes over time. A further limitation was that we were not able to assess the potential region difference (urban versus rural) in associations of active commuting with cardiovascular outcomes because of a lack of information among farmers. In addition, our results primarily concern middle‐aged and older commuters living in urban cities. The applicability of our findings to other age and racial/ethnic groups needs to be addressed in further research. Finally, although we have adjusted for multiple confounding factors, residual confounding, particularly socioeconomic status, was still possible. However, active commuting was associated with low socioeconomic status in this study population.

## Conclusions

In summary, our study is the first and thus far the largest prospective investigation on the long‐term associations between active commuting and CVDs in Chinese (Asians). We found that daily cycling to and from work is related to a reduced risk of IHD and IS in urban China. Walking was associated with a lower risk of IHD. Our findings provide direct support for policies that encourage adults to participate in active commuting to deliver health benefits at the population level.

## Sources of Funding

This work was supported by grants (2016YFC0900500, 2016YFC0900501, 2016YFC0900504) from the National Key R&D Program of China. The CKB baseline survey and the first resurvey were supported by a grant from the Kadoorie Charitable Foundation in Hong Kong. The long‐term follow‐up is supported by grants from the UK Wellcome Trust (202922/Z/16/Z, 088158/Z/09/Z, 104085/Z/14/Z), National Natural Science Foundation of China (81390540, 81390544, 81390541), and Chinese Ministry of Science and Technology (2011BAI09B01). Dr Qi is supported by grants from the National Heart, Lung, and Blood Institute (HL071981, HL034594, HL126024) and the National Institute of Diabetes and Digestive and Kidney Diseases (DK115679, DK091718, DK100383, DK078616). Fan is a recipient of a scholarship under the China Scholarship Council to pursue her study in the United States of America (201706010313). The funders had no role in the study design, data collection, data analysis and interpretation, writing of the report, or the decision to submit the article for publication.

## Disclosures

None.

## Supporting information


**Appendix S1.** Members of the China Kadoorie Biobank Collaborative Group.
**Table S1.** Adjusted Hazard Ratios for Cardiovascular Diseases by Baseline Commuting Mode
**Table S2.** Subgroup Analysis of Associations Between Commuting Mode and Ischemic Heart Disease According to Potential Baseline Risk Factors
**Table S3.** Subgroup Analysis of Associations Between Commuting Mode and Ischemic Stroke According to Potential Baseline Risk Factors
**Table S4.** Subgroup Analysis of Associations Between Commuting Mode and Hemorrhagic Stroke According to Potential Baseline Risk Factors
