# Supplementary material for: Association Between Active Commuting and Incident Cardiovascular Diseases in Chinese: A Prospective Cohort Study
Source: J Am Heart Assoc. 2019 Oct 2;8(20):e012556. doi: 10.1161/JAHA.119.012556 (PMC6818036; doi:10.1161/JAHA.119.012556)
Supplement: Supplementary file 1 — Appendix S1. Members of the China Kadoorie Biobank Collaborative Group. Table S1. Adjusted Hazard Ratios for Cardiovascular Diseases by Baseline Commuting Mode Table S2. Subgroup Analysis of Associations Between Commuting Mode and Ischemic Heart Disease According to Potential Baseline Risk Factors Table S3. Subgroup Analysis of Associations Between Commuting Mode and Ischemic Stroke According to Potential Baseline Risk Factors Table S4. Subgroup Analysis of Associations Between Commuting Mode and Hemorrhagic Stroke According to Potential Baseline Risk Factors [file JAH3-8-e012556-s001.pdf]

Supplemental Material

Appendix

Members of the China Kadoorie Biobank collaborative group:

International Steering Committee: Junshi Chen, Zhengming Chen (PI), Robert Clarke, Rory Collins, Yu Guo, Liming Li (PI), Jun Lv, Richard Peto, Robin Walters. **International Co-ordinating Centre, Oxford:** Daniel Avery, Ruth Boxall, Derrick Bennett, Yumei Chang, Yiping Chen, Zhengming Chen, Robert Clarke, Huaidong Du, Simon Gilbert, Alex Hacker, Mike Hill, Michael Holmes, Andri Iona, Christiana Kartsonaki, Rene Kerosi, Ling Kong, Om Kurmi, Garry Lancaster, Sarah Lewington, Kuang Lin, John McDonnell, Iona Millwood, Qunhua Nie, Jayakrishnan Radhakrishnan, Paul Ryder, Sam Sansome, Dan Schmidt, Paul Sherliker, Rajani Sohoni, Becky Stevens, Iain Turnbull, Robin Walters, Jenny Wang, Lin Wang, Neil Wright, Ling Yang, Xiaoming Yang. **National Co-ordinating Centre, Beijing:** Zheng Bian, Yu Guo, Xiao Han, Can Hou, Jun Lv, Pei Pei, Chao Liu, Yunlong Tan, Canqing Yu. **10 Regional Co-ordinating Centres: Qingdao CDC:** Zengchang Pang, Ruqin Gao, Shanpeng Li, Shaojie Wang, Yongmei Liu, Ranran Du, Yajing Zang, Liang Cheng, Xiaocao Tian, Hua Zhang, Yaoming Zhai, Feng Ning, Xiaohui Sun, Feifei Li. **Licang CDC:** Silu Lv, Junzheng Wang, Wei Hou. **Heilongjiang Provincial CDC:** Mingyuan Zeng, Ge Jiang, Xue Zhou. **Nangang CDC:** Liqiu Yang, Hui He, Bo Yu, Yanjie Li, Qinai Xu,Quan Kang, Ziyan Guo. **Hainan Provincial CDC:** Dan Wang, Ximin Hu, Jinyan Chen, Yan Fu, Zhenwang Fu, Xiaohuan Wang. **Meilan CDC:** Min Weng, Zhendong Guo, Shukuan Wu,Yilei Li, Huimei Li, Zhifang Fu. **Jiangsu Provincial CDC:** Ming Wu, Yonglin Zhou, Jinyi Zhou, Ran Tao, Jie Yang, Jian Su. **Suzhou CDC:** Fang liu, Jun Zhang, Yihe Hu, Yan Lu, , Liangcai Ma, Aiyu Tang, Shuo Zhang, Jianrong Jin, Jingchao Liu. **Guangxi Provincial CDC:** Zhenzhu Tang, Naying Chen, Ying Huang. **Liuzhou CDC:** Mingqiang Li, Jinhuai Meng, Rong Pan, Qilian Jiang, Jian Lan,Yun Liu, Liuping Wei, Liyuan Zhou, Ningyu Chen Ping Wang, Fanwen Meng, Yulu Qin,, Sisi Wang. **Sichuan Provincial CDC:** Xianping Wu, Ningmei Zhang, Xiaofang Chen,Weiwei Zhou. **Pengzhou CDC:** Guojin Luo, Jianguo Li, Xiaofang Chen, Xunfu Zhong, Jiaqiu Liu, Qiang Sun. **Gansu Provincial CDC:** Pengfei Ge, Xiaolan Ren, Caixia Dong. **Maiji CDC:** Hui Zhang, Enke Mao, Xiaoping Wang, Tao Wang, Xi zhang. **Henan Provincial CDC:** Ding Zhang, Gang Zhou, Shixian Feng, Liang Chang, Lei Fan. **Huixian CDC:** Yulian Gao, Tianyou He, Huarong Sun, Pan He, Chen Hu, Xukui Zhang, Huifang Wu, Pan He. **Zhejiang Provincial CDC:** Min Yu, Ruying Hu, Hao Wang. Tongxiang CDC: Yijian Qian, Chunmei Wang, Kaixu Xie, Lingli Chen, Yidan Zhang, Dongxia Pan, Qijun Gu. **Hunan Provincial CDC:** Yuelong Huang, Biyun Chen, Li Yin, Huilin Liu, Zhongxi Fu, Qiaohua Xu. **Liuyang CDC:** Xin Xu, Hao Zhang, Huajun Long, Xianzhi Li, Libo Zhang, Zhe Qiu.

Table S1. Adjusted Hazard ratios for cardiovascular diseases by baseline commuting mode.

	N	Ischemic heart disease	Ischemic stroke	Hemorrhagic stroke
Car or public transport	28,242	[Reference]	[Reference]	[Reference]
Motorcycle or moped	20,903	1.04 (0.92,1.17)	1.08 (0.95,1.22)	1.24 (0.93,1.66)
Work at home or work near home	13,936	**0.91 (0.83,1.00)**	1.06 (0.96,1.16)	1.28 (0.98,1.68)
Walking	20,912	**0.90 (0.84,0.97)**	1.06 (0.98,1.15)	0.96 (0.75,1.23)
Cycling	20,177	**0.82 (0.75,0.89)**	0.94 (0.85,1.03)	1.12 (0.87,1.44)

Stratified Cox proportional models were used with stratification on age and study area. Multivariable model was adjusted for sex; education; marital status; household income; occupation; alcohol consumption; smoking status; intake frequencies of red meat, fresh fruits, and vegetables; leisure sedentary time; family history of heart attack or stroke (only in the corresponding analysis); body mass index; prevalent hypertension; prevalent diabetes; cooking pollution; heating pollution; passive smoking; occupational, housework, and leisure-time physical activity level.

Table S2. Subgroup analysis of associations between commuting mode and ischemic heart disease according to potential baseline risk factors.

	Work at home/near home		Walking		Cycling		$P_{interaction}$ *
	HR (95% CI)	P†	HR (95% CI)	P†	HR (95% CI)	P†	
All	0.90 (0.82,0.99)		0.90 (0.84,0.96)		0.81 (0.74,0.88)		
Sex							0.428
men	0.91 (0.81,1.03)		0.92 (0.84,1.01)		0.86 (0.77,0.95)		
women	0.93 (0.81,1.06)	0.778	0.91 (0.82,1.00)	0.946	0.80 (0.69,0.93)	0.109	
Age at baseline							0.651
<50 y	0.90 (0.79,1.02)		0.88 (0.81,0.96)		0.77 (0.68,0.86)		
≥50 y	0.88 (0.77,1.00)	0.816	0.90 (0.80,1.00)	0.430	0.85 (0.75,0.97)	0.237	
Education level							0.252
Illiterate/primary school	0.78 (0.59,1.03)		0.73 (0.55,0.98)		0.70 (0.54,0.91)		
Middle school and above	0.92 (0.83,1.01)	0.087	0.90 (0.84,0.97)	0.108	0.82 (0.74,0.89)	0.141	
Marital status							0.033
Unmarried	0.62 (0.45,0.85)		0.87 (0.69,1.10)		0.74 (0.54,1.01)		
Married	0.94 (0.85,1.03)	0.004	0.90 (0.84,0.97)	0.461	0.81 (0.75,0.89)	0.375	
Household income							0.511
<20000/year	0.92 (0.79,1.07)		0.94 (0.83,1.05)		0.88 (0.77,1.01)		
>20000/year	0.90 (0.80,1.00)	0.847	0.88 (0.81,0.95)	0.739	0.76 (0.68,0.85)	0.134	
Occupation							0.810
Non-factory worker	0.91 (0.82,1.01)		0.92 (0.85,1.01)		0.82 (0.72,0.93)		
Factory worker	0.88 (0.73,1.05)	0.858	0.86 (0.77,0.95)	0.370	0.79 (0.70,0.89)	0.908	
Smoking status							0.317
Not current daily smoker	0.96 (0.85,1.07)		0.90 (0.82,0.98)		0.82 (0.73,0.91)		
Current daily smoker	0.85 (0.73,0.98)	0.078	0.92 (0.82,1.03)	0.768	0.83 (0.73,0.94)	0.838	
Alcohol consumption							0.896
Not current weekly drinker	0.88 (0.79,0.97)		0.89 (0.82,0.97)		0.78 (0.71,0.87)		
Current weekly drinker	0.97 (0.82,1.15)	0.728	0.91 (0.80,1.03)	0.833	0.88 (0.76,1.02)	0.551	
Other physical activity level							0.894
Q1	0.77 (0.61,0.98)		0.92 (0.77,1.11)		0.77 (0.59,1.02)		
Q2	0.90 (0.79,1.03)	0.372	0.87 (0.79,0.96)	0.711	0.79 (0.69,0.91)	0.815	
Q3	0.97 (0.84,1.13)	0.202	0.92 (0.82,1.02)	0.982	0.84 (0.74,0.94)	0.590	
Leisure sedentary time							0.412
<3 h/day	0.91 (0.80,1.04)		0.85 (0.77,0.94)		0.76 (0.68,0.87)		
≥3 h/day	0.89 (0.79,1.01)	0.519	0.95 (0.86,1.04)	0.254	0.85 (0.75,0.95)	0.378	
Body mass index							0.806
<24	0.91 (0.78,1.05)		0.91 (0.82,1.02)		0.81 (0.71,0.92)		
24-<28	0.83 (0.72,0.96)	0.293	0.88 (0.79,0.98)	0.563	0.78 (0.69,0.89)	0.722	
≥28	1.06 (0.87,1.30)	0.688	0.88 (0.74,1.03)	0.486	0.88 (0.72,1.08)	0.699	
Hypertension							0.109

	Work at home/near home		Walking		Cycling		$P_{interaction}$ *
	HR (95% CI)	P†	HR (95% CI)	P†	HR (95% CI)	P†	
No	0.88 (0.78,0.99)		0.92 (0.85,1.00)		0.77 (0.69,0.86)		
Yes	0.95 (0.82,1.09)	0.653	0.86 (0.77,0.97)	0.075	0.87 (0.76,0.99)	0.274	
Diabetes							0.299
No	0.90 (0.82,0.99)		0.91 (0.85,0.98)		0.81 (0.74,0.89)		
Yes	0.89 (0.68,1.18)	0.684	0.79 (0.63,0.99)	0.061	0.80 (0.60,1.06)	0.811	

The reference category for all analyses was non-active commuting. Stratified Cox models were used and analyses were done separately for each baseline characteristic. Models were adjusted for sex; education; marital status; household income; occupation; alcohol consumption; smoking status; intake frequencies of red meat, fresh fruits, and vegetables; leisure sedentary time; family history of heart attack; body mass index; prevalent hypertension; prevalent diabetes; cooking pollution; heating pollution; passive smoking; occupational, housework, and leisure-time physical activity level, except for the stratified variable in the corresponding subgroup analysis.

* P interaction: the tests for interaction were performed by means of likelihood ratio tests, which involved comparing models with and without cross product terms between the baseline characteristic and commuting mode (4 categories).

† P value of the Wald test.

Table S3. Subgroup analysis of associations between commuting mode and ischemic stroke according to potential baseline risk factors.

	Work at home/near home		Walking		Cycling		$P_{interaction}$ *
	HR (95% CI)	P†	HR (95% CI)	P†	HR (95% CI)	P†	
All	1.04 (0.95,1.13)		1.05 (0.97,1.13)		**0.92 (0.84,1.00)**		
Sex							0.789
men	1.06 (0.94,1.18)		1.07 (0.98,1.18)		0.93 (0.84,1.03)		
women	1.04 (0.90,1.20)	0.634	1.03 (0.92,1.16)	0.655	0.96 (0.82,1.12)	0.575	
Age at baseline							0.203
<50 y	1.10 (0.96,1.25)		1.07 (0.97,1.19)		0.88 (0.78,1.00)		
≥50 y	0.95 (0.84,1.07)	0.189	0.97 (0.87,1.08)	0.432	0.90 (0.80,1.01)	0.317	
Education level							0.137
Illiterate/primary school	0.87 (0.70,1.09)		0.95 (0.75,1.20)		0.77 (0.62,0.94)		
Middle school and above	1.08 (0.98,1.19)	0.028	1.06 (0.98,1.14)	0.396	0.95 (0.86,1.04)	0.077	
Marital status							0.564
Unmarried	1.25 (0.92,1.70)		1.13 (0.87,1.48)		0.86 (0.60,1.21)		
Married	1.02 (0.93,1.12)	0.862	1.04 (0.96,1.12)	0.559	0.92 (0.84,1.01)	0.344	
Household income							0.155
<20000/year	1.05 (0.91,1.21)		1.14 (1.01,1.28)		0.96 (0.84,1.10)		
>20000/year	1.04 (0.93,1.16)	0.730	1.00 (0.91,1.10)	0.059	0.89 (0.79,0.99)	0.342	
Occupation							0.316
Non-factory worker	1.08 (0.97,1.20)		1.05 (0.96,1.16)		1.01 (0.89,1.14)		
Factory worker	1.01 (0.85,1.20)	0.345	1.06 (0.95,1.19)	0.858	0.86 (0.76,0.97)	0.085	
Smoking status							0.909
Not current daily smoker	1.03 (0.91,1.15)		1.05 (0.95,1.16)		0.92 (0.82,1.04)		
Current daily smoker	1.08 (0.94,1.24)	0.651	1.07 (0.96,1.20)	0.890	0.94 (0.83,1.07)	0.694	
Alcohol consumption							0.221
Not current weekly drinker	1.01 (0.91,1.13)		0.99 (0.90,1.09)		0.93 (0.84,1.04)		
Current weekly drinker	1.10 (0.94,1.28)	0.647	1.16 (1.03,1.31)	0.066	0.91 (0.79,1.04)	0.757	
Other physical activity level							0.905
Q1	0.95 (0.75,1.20)		1.10 (0.90,1.34)		0.87 (0.66,1.16)		
Q2	1.07 (0.93,1.22)	0.709	1.01 (0.90,1.13)	0.415	0.91 (0.79,1.04)	0.906	
Q3	1.02 (0.89,1.17)	0.885	1.08 (0.96,1.21)	0.740	0.94 (0.83,1.05)	0.973	
Leisure sedentary time							0.736
<3 h/day	1.08 (0.95,1.23)		1.07 (0.97,1.19)		0.93 (0.82,1.05)		
≥3 h/day	0.99 (0.88,1.12)	0.347	1.03 (0.93,1.14)	0.572	0.91 (0.81,1.03)	0.875	
Body mass index							0.373
<24	1.08 (0.94,1.25)		1.04 (0.92,1.17)		0.87 (0.76,1.00)		
24-<28	0.96 (0.84,1.10)	0.194	1.09 (0.97,1.21)	0.498	0.93 (0.82,1.06)	0.303	
≥28	1.12 (0.91,1.38)	0.670	0.98 (0.81,1.17)	0.429	0.97 (0.79,1.21)	0.693	
Hypertension							0.251

	Work at home/near home		Walking		Cycling		$P_{interaction}$ *
	HR (95% CI)	P†	HR (95% CI)	P†	HR (95% CI)	P†	
No	1.04 (0.92,1.17)		1.00 (0.91,1.11)		0.89 (0.79,1.00)		
Yes	1.06 (0.93,1.20)	0.252	1.12 (1.00,1.25)	0.466	0.96 (0.85,1.09)	0.323	
Diabetes							0.374
No	1.01 (0.92,1.11)		1.04 (0.96,1.13)		0.91 (0.83,1.00)		
Yes	1.32 (1.02,1.71)	0.168	1.13 (0.90,1.41)	0.832	1.01 (0.78,1.30)	0.319	

The same as in Table S2.

Table S4. Subgroup analysis of associations between commuting mode and hemorrhagic stroke according to potential baseline risk factors.

	Work at home/near home		Walking		Cycling		$P_{interaction}$ *
	HR (95% CI)	P†	HR (95% CI)	P†	HR (95% CI)	P†	
All	1.17 (0.92,1.49)		0.89 (0.71,1.11)		1.01 (0.82,1.26)		
Sex							0.432
men	1.25 (0.95,1.65)		0.91 (0.70,1.18)		1.06 (0.83,1.35)		
women	1.06 (0.66,1.70)	0.237	0.83 (0.54,1.27)	0.464	0.83 (0.52,1.32)	0.127	
Age at baseline							0.131
<50 y	1.35 (0.96,1.91)		0.93 (0.70,1.25)		1.16 (0.86,1.56)		
≥50 y	0.97 (0.70,1.34)	0.079	0.74 (0.52,1.04)	0.125	0.80 (0.59,1.09)	0.046	
Education level							0.760
Illiterate/primary school	1.23 (0.79,1.93)		0.73 (0.41,1.31)		0.90 (0.60,1.36)		
Middle school and above	1.17 (0.88,1.56)	0.956	0.93 (0.73,1.19)	0.360	1.06 (0.82,1.36)	0.529	
Marital status							0.359
Unmarried	1.91 (0.71,5.14)		1.74 (0.76,3.98)		1.45 (0.57,3.69)		
Married	1.14 (0.89,1.46)	0.378	0.84 (0.66,1.06)	0.075	0.98 (0.79,1.22)	0.397	
Household income							0.830
<20000/year	1.05 (0.72,1.54)		0.85 (0.60,1.22)		1.05 (0.75,1.46)		
>20000/year	1.22 (0.90,1.67)	0.484	0.91 (0.69,1.22)	0.612	0.95 (0.71,1.26)	0.871	
Occupation							0.977
Non-factory worker	1.18 (0.88,1.59)		0.90 (0.66,1.23)		0.97 (0.70,1.36)		
Factory worker	1.24 (0.81,1.91)	0.909	0.88 (0.64,1.20)	0.950	1.05 (0.79,1.39)	0.693	
Smoking status							0.879
Not current daily smoker	1.19 (0.84,1.69)		0.94 (0.68,1.28)		1.01 (0.73,1.39)		
Current daily smoker	1.22 (0.88,1.69)	0.560	0.83 (0.60,1.13)	0.885	0.99 (0.75,1.32)	0.593	
Alcohol consumption							0.523
Not current weekly drinker	1.30 (0.97,1.75)		0.97 (0.73,1.29)		1.01 (0.76,1.34)		
Current weekly drinker	1.05 (0.70,1.58)	0.310	0.74 (0.51,1.06)	0.214	1.02 (0.73,1.42)	0.885	
Other physical activity level							0.456
Q1	1.05 (0.47,2.32)		0.72 (0.33,1.57)		1.58 (0.71,3.50)		
Q2	1.30 (0.87,1.93)	0.783	1.12 (0.80,1.59)	0.520	1.03 (0.69,1.55)	0.275	
Q3	1.08 (0.77,1.51)	0.947	0.75 (0.55,1.04)	0.668	0.94 (0.72,1.23)	0.137	
Leisure sedentary time							0.585
<3 h/day	1.39 (0.99,1.95)		0.87 (0.63,1.20)		0.96 (0.70,1.31)		
≥3 h/day	1.00 (0.71,1.40)	0.304	0.91 (0.67,1.23)	0.882	1.09 (0.81,1.46)	0.623	
Body mass index							0.639
<24	1.16 (0.80,1.67)		0.78 (0.54,1.12)		1.00 (0.73,1.37)		
24-<28	1.16 (0.79,1.68)	0.754	0.86 (0.61,1.21)	0.494	0.93 (0.66,1.31)	0.842	
≥28	1.36 (0.76,2.44)	0.697	1.35 (0.81,2.26)	0.062	1.39 (0.80,2.39)	0.306	
Hypertension							0.075

	Work at home/near home		Walking		Cycling		$P_{interaction}$ *
	HR (95% CI)	P†	HR (95% CI)	P†	HR (95% CI)	P†	
No	1.25 (0.83,1.90)		0.65 (0.43,1.01)		0.84 (0.57,1.24)		
Yes	1.11 (0.83,1.49)	0.162	0.99 (0.76,1.28)	0.092	1.07 (0.83,1.38)	0.889	
Diabetes							0.994
No	1.18 (0.91,1.52)		0.90 (0.71,1.14)		1.03 (0.82,1.29)		
Yes	1.07 (0.53,2.16)	0.903	0.87 (0.45,1.66)	0.841	0.90 (0.47,1.73)	0.994	

The same as in Table S2.
